# Radiofrequency ablation in an infant with recurrent supraventricular tachycardia and cyanosis

**DOI:** 10.4103/0974-2069.58319

**Published:** 2009

**Authors:** Amit Vora, Yash Lokhandwala, Chirag Sheth, Bharat Dalvi

**Affiliations:** Arrhythmia Associates, Mumbai, India; 1 Glenmark Cardiac Centre, Mumbai, India

**Keywords:** Electrophysiology study, arrhythmias, anti-arrhyhmic drugs

## Abstract

We report an unusual presentation of supraventricular tachycardia, in an infant, with cyanosis. The child had atrial septal defect with hypoplastic right ventricle. Radiofrequency ablation was performed in view of drug resistant SVT

## INTRODUCTION

Cardiac arrhythmias are relatively uncommon in the younger population, accounting for approximately 5% of all emergency cardiac admissions.[1] Paroxysmal supraventricular tachycardia (SVT) like atrio-ventricular reciprocating tachycardia (AVRT), ectopic atrial tachycardia, and atrio-ventricular nodal reentrant tachycardia are the most common. In those patients who have undergone operations for congenital heart disease, junctional ectopic tachycardia and atrial flutter are more common.[1] Symptoms of arrhythmias depend on the age, presence of structural heart disease, and the state of the left ventricular function apart from the nature, severity, and duration of arrhythmia. In newborns and infants, tachyarrhythmias present with shortness of breath, increased precordial activity, listlessness, irritability, refusal to feed, or convulsions. We report a case of a recurrent SVT uniquely presenting with cyanosis.

## CASE REPORT

A 10-month-old male born at full term of non-consanguineous marriage presented with episodic listlessness associated with bluish discoloration of the lips. He had recurrent respiratory tract infections and a failure to thrive.

**Figure 1:**
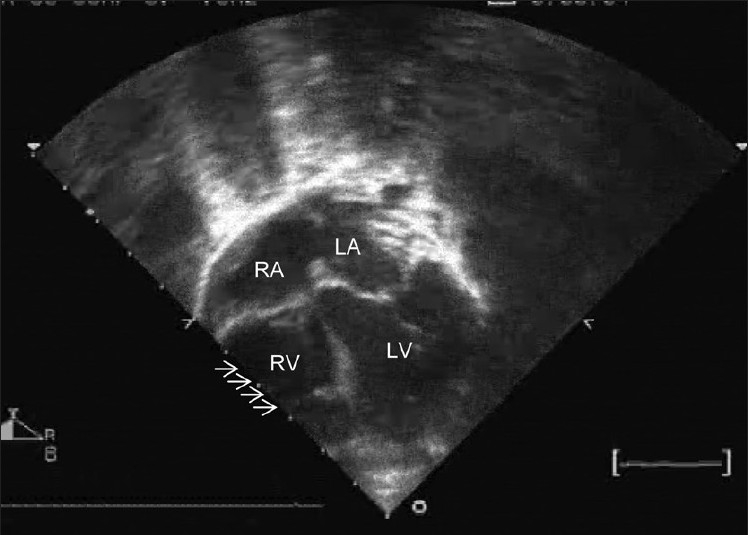
Echocardiogram showing a large ASD with hypoplastic right ventricle (marked with arrows)

During one such episode, the pediatrician noted rapid heart beats and an ECG revealed narrow QRS tachycardia at the rate of 260 beats per minute. Oxygen saturation was 80% on room air. Adenosine terminated the tachycardia. A post-termination ECG in sinus rhythm did not reveal preexcitation. A clinical examination during sinus rhythm revealed an absence of cyanosis (O_2_ saturation of 95% at rest); a normal-sized heart; quiet precordium, and a Grade II/VI short ejection systolic murmur in the left parasternal region. A chest X-ray showed mild cardiomegaly with normal vascularity. An echocardiography revealed a large ostium secundum atrial septal defect (ASD) with bidirectional shunt. There was no pulmonic stenosis or pulmonary hypertension. However, the right ventricle was hypoplastic [Figure 1] with tricuspid valve annulus measuring 10.5 mm while the mitral annulus measured 16 mm. The right atrium was enlarged. The left atrium, left ventricle, and mitral and aortic valves were normal.

To prevent recurrences of SVT, digoxin was started. However, SVT continued to recur and propranolol was added. Despite this combination, there were breakthroughs and eventually amiodarone was used. On 5 mg/kg/day of amiodarone, there was a recurrence of SVT requiring hospitalization. It was therefore decided to subject the patient to an electrophysiology (EP) study with a view to radiofrequency (RF) ablation. Amiodarone was omitted for 10 days prior to the EP study. At the time of the procedure, the child was 1 year old and weighed 7.2 kg. The procedure was performed under general anesthesia.

**Figure 2:**
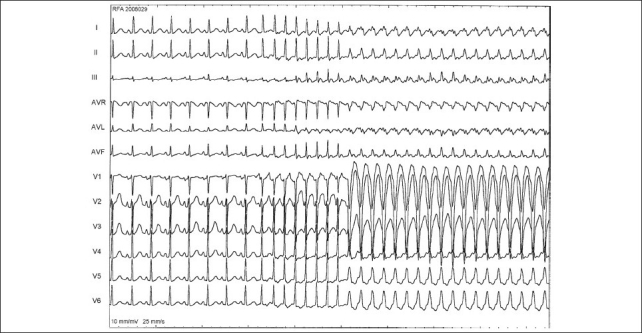
12 lead ECG. Sinus rhythm show no preexcitation. At the beginning of the SVT there is narrow QRS and subsequently tachycardia continues with LBBB. Note the slowing of tachycardia with LBBB, suggesting left sided accessory pathway being used during the orthodromic atrio-ventricular reentrant tachycardia

A 5F sheath was introduced in the left femoral vein through which a 5F deflectable decapolar catheter was introduced in the coronary sinus. A 4F sheath was used in the right femoral vein and a 4F quadripolar catheter was introduced in the bundle region. The HV interval was normal. The ventriculo-atrial (VA) conduction was eccentric via a concealed left lateral accessory pathway (AP). An orthodromic AVRT [Figure 2] could be reproducibly induced with ventricular extrastimuli. There was a spontaneous appearance of left bundle branch block during tachycardia, when the tachycardia cycle length slowed by 20 ms further establishing a left-sided AP mediated orthodromic AVRT [Figure 2].

**Figure 3:**
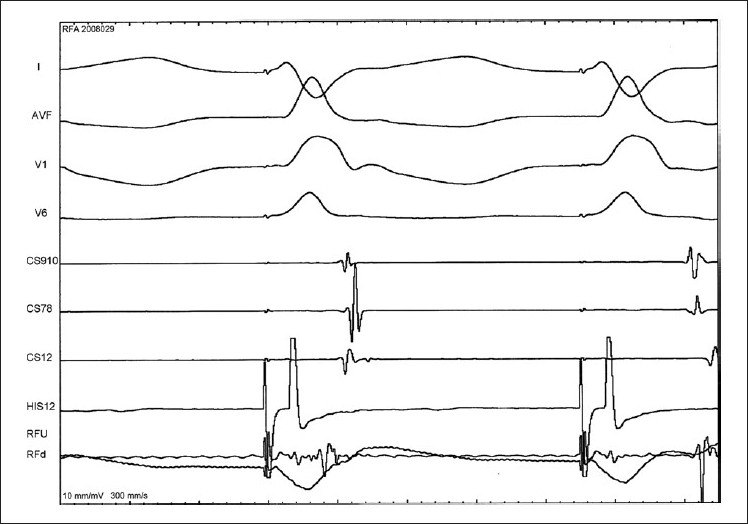
Surface ECG leads I, AVF, V1 and V6 with intracardiac electrograms during successful radiofrequency ablation of the left lateral accessory pathway. Ablation is performed during left ventricular pacing and intracardia electrograms reveal shift of activation from distal CS early to proximal CS early and separation of the local ventriculo atrial in the radiofrequency catheter

**Figure 4:**
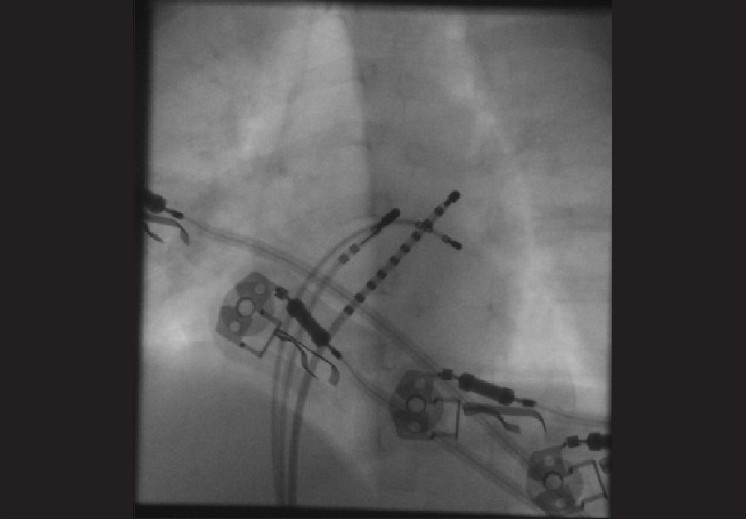
Fluroscopy image in 40 degree LAO view showing the decapolar CS catheter. Quadripolar catheter in the LV and the radiofrequency ablation catheter on the mitral annulus in the left postero-lateral region where successful RF ablation was performed

An additional 5F sheath was introduced in the right femoral vein. A 5F radiofrequency ablation catheter was used. Mapping was performed during left ventricular pacing (via the ASD) using the quadripolar catheter. The mitral annulus was mapped and the site of AP identified. Radiofrequency energy was applied in the temperature controlled mode with a cut-off of 60 degrees centigrade and power limit of 25 watts. Successful RF ablation [Figure 3] was performed immediately with the first lesion in the left postero-lateral region [Figure 4]. After ablation, there was VA conduction over the atrioventricular (AV) node; no SVT was inducible and there was no evidence of AP. On a 6-month follow-up visit, there was no recurrence of SVT.

## DISCUSSION

Accessory pathway mediated tachycardias constitute 80% of all the tachyarrhythmias during infancy. In some infants they resolve as the child grows.[2] Most SVTs beyond the age of 5 tend to recur and may need to undergo RF ablation. Ectopic atrial tachycardia and atrial flutter represent 10-15% of SVTs in infancy, of which some may not ever recur.

Radiofrequency ablation is uncommonly performed under 5 years of age. This is because some SVTs resolve spontaneously. Also, arrhythmias may get suppressed under the influence of anesthesia at the time of RF ablation. Patient size poses limitations in using multiple electrode catheters and there is an increased possibility of cardiac/valve damage with ablation. The risks of ablation are acceptable only if the child has recurrent, drug-resistant SVT especially if it results in tachycardiomyopathy or life-threatening cardiovascular compromise.[3‐8]

Our case of SVT is unique in its presentation with cyanosis. This 1-year-old child had recurrent, drug-resistant SVT. The large ASD and hypoplastic RV resulted in hypoxia during SVT. The right to left shunt may have increased during rapid heart rates because of the shortening of diastole and increased trans-tricuspid gradient-almost similar to what one sees during tachycardia in mitral stenosis. Propafenone or flecainide are probably more effective agents for managing these tachycardias; however, these are not easily available in India. Adenosine responsiveness of the tachycardia suggested AV node dependant SVT and therefore is more likely to be successfully ablated. The large ASD provided easy access to the left heart for ablation without the need for arterial access.

Left-sided accessory pathways has been accessed by the antegrade (via the inter-atrial septum) or the retrograde trans-aortic route (via the aorta and left ventricle). In young children, the antegrade approach is preferred to avoid damage to the aortic/mitral valves and also avoiding arterial access. Radiofrequency ablation by the antegrade approach was facilitated in our patient because of the ASD. Tackling the SVT before ASD closure in patients with left-sided AP avoids the inconvenience of a septal puncture through the ASD patch. Mapping and RF ablation was performed in our patient during LV pacing. The latter allowed VA conduction exclusively over the left lateral AP.

The current case highlights a rather unusual presentation of SVT with cyanosis. The resistant nature of the tachycardia and non availability of a certain group of anti-arrhythmics prompted us to undertake RF ablation in this young child. The presence of an ASD certainly facilitated the approach to the AP located on the left lateral wall.
